# A thermostable, chromatographically purified Ebola nano-VLP vaccine

**DOI:** 10.1186/s12967-015-0593-y

**Published:** 2015-07-15

**Authors:** John H Carra, Karen A O Martins, Rowena D Schokman, Camenzind G Robinson, Jesse T Steffens, Sina Bavari

**Affiliations:** Molecular and Translational Sciences, United States Army Medical Research Institute of Infectious Diseases, Fort Detrick, MD 21702-9211 USA; Pathology Division, United States Army Medical Research Institute of Infectious Diseases, Fort Detrick, MD 21702-9211 USA; Janelia Farm Research Campus, Howard Hughes Medical Institute, Ashburn, VA 20147 USA

**Keywords:** Filovirus, vaccine, Ebola, Virus-like particle, Immunogenicity, Thermostability

## Abstract

**Background:**

Filovirus virus-like particles (VLP) are strong immunogens with the potential for development into a safe, non-infectious vaccine. However, the large size and filamentous structure of this virus has heretofore made production of such a vaccine difficult. Herein, we present new assays and a purification procedure to yield a better characterized and more stable product.

**Methods:**

Sonication of VLP was used to produce smaller “nano-VLP”, which were purified by membrane chromatography. The sizes and lengths of VLP particles were analyzed using electron microscopy and an assay based on transient occlusion of a nanopore. Using conformationally-sensitive antibodies, we developed an in vitro assay for measuring GP conformational integrity in the context of VLP, and used it to profile thermal stability.

**Results:**

We developed a new procedure for rapid isolation of Ebola VLP using membrane chromatography that yields a filterable and immunogenic product. Disruption of VLP filaments by sonication followed by filtration produced smaller particles of more uniform size, having a mean diameter close to 230 nm. These reduced-size VLP retained GP conformation and were protective against mouse-adapted Ebola challenge in mice. The “nano-VLP” consists of GP-coated particles in a mixture of morphologies including circular, branched, “6”-shaped, and filamentous ones up to ~1,500 nm in length. Lyophilization conferred a high level of thermostability on the nano-VLP. Unlike Ebola VLP in solution, which underwent denaturation of GP upon moderate heating, the lyophilized nano-VLP can withstand at least 1 h at 75°C, while retaining conformational integrity of GP and the ability to confer protective immunity in a mouse model.

**Conclusions:**

We showed that Ebola virus-like particles can be reduced in size to a more amenable range for manipulation, and that these smaller particles retained their temperature stability, the structure of the GP antigen, and the ability to stimulate a protective immune response in mice. We developed a new purification scheme for “nano-VLP” that is more easily scaled up and filterable. The product could also be made thermostable by lyophilization, which is highly significant for vaccines used in tropical countries without a reliable “cold-chain” of refrigeration.

**Electronic supplementary material:**

The online version of this article (doi:10.1186/s12967-015-0593-y) contains supplementary material, which is available to authorized users.

## Background

The filoviruses Ebola and Marburg are enveloped viruses causing lethal, hemorrhagic disease in humans and non-human primates [1]. The virions exist in a mixture of morphologies, including “6”-shaped and filamentous particles. The filaments are 80–100 nm in width and can be several microns long [2]. The surface of the virions is covered in trimeric spikes of the glycoprotein (GP), while the VP40 protein forms a structural matrix underlying the viral membrane. Formation of virus-like particles (VLP) with shapes similar to authentic filoviruses can be induced by transfection into human or insect cell lines of the genes for GP and VP40 alone [3–6]. The five other viral proteins are not essential for production of virus-like particles, although some efforts have also included the nucleocapsid protein NP.

VLP are useful as laboratory reagents for the exploration of filovirus biology, and they are promising candidates for vaccines to protect humans against natural or deliberate exposure to these viruses [7]. Non-human primates have been successfully immunized against Ebola with VLP plus adjuvant, with at least two doses needed for full protection, while one conferred partial protection [8, 9]. Filovirus VLP are more effective than soluble GP proteins at stimulating the immune system [10]. The presentation of GP trimers in a repetitive array likely contributes to their potency by increasing interactions with receptors on B cells and antigen-presenting cells [2, 11]. However, unlike VLP of some other types of viruses, which may consist only of smaller, spherical and non-enveloped proteinaceous particles that can be reassembled in vitro from isolated subunits, filovirus VLP can be several microns in length and are enveloped. These characteristics present difficult problems in purification, sterilization and analytical methods. Development of the filovirus VLP as vaccines for humans hence has been limited by the methods used in production, which rely upon sucrose gradient ultracentrifugation for purification and gamma-irradiation for sterilization. Production of the VLP by transient transfection also introduces high costs to supply the doses used in NHP experiments (typically 50–250 µg GP), therefore more efficient means of expression are desirable.

The influence of the large size of the filaments on stimulation of the immune response is not yet clear. The optimal size and shape of nanoparticle vaccines is an important factor in their design and a subject of current interest (reviewed in [12–14]). For example, Manolova et al. [15] studied the effect of particle size on antigen uptake by dendritic cells and found that polystyrene beads of ≤200 nm were able to drain rapidly to lymph nodes, where they were taken up by lymph node-resident dendritic cells and macrophages. Beads ≥500 nm could not directly enter the lymphatic system, and were taken up more slowly by a different population of dendritic cells at the site of injection. Champion and Mitragotri [16] found that worm-like polystyrene particles were taken up by macrophages very poorly when compared to spherical particles. These results suggest that it may be advantageous to the immune response to reduce the length of filovirus VLP.

In this study, we examined how changes in particle size and GP conformation affect immunogenicity using a combination of mouse bioassays, electron microscopy (EM), antibody-based probes of GP, and a nanopore sizing method. We also demonstrate a rapid in vitro test for antigenic integrity that was correlated to in vivo potency, and confirm the functional significance of conformationally intact GP for the vaccine. We present results describing a new version of the Ebola VLP vaccine consisting of smaller particles that are more easily purified and can be filtered to reduce bioburden.

## Methods

### VLP production and purification

VLPs were produced at NCI and USAMRIID using a modification of the procedure described by Warfield et al. [3]. In brief, VLPs were created by transfecting HEK 293 cells with expression vectors containing the genes for GP and VP40 proteins. After a low-speed centrifugation to remove cells, VLP in the culture supernatant were centrifuged to a pellet using a Beckman JLA10.5 rotor at 10,000 rpm (~18,500×*g*). The pellet was resuspended in PBS and applied to a 10–60% sucrose gradient. The gradient was then spun at 174,000×*g* for 14 h. The resulting band was removed from the gradient and washed twice in sterile PBS. The VLP were resuspended in PBS and stored at 4°C, to be used in freeze/thaw studies. VLP were also produced under a contract at Paragon Bioservices, using a sucrose-gradient based method performed at a larger scale with a Wave bioreactor and HEK 293F suspension cells. These samples were stored frozen at −80°C.

The concentration of GP in the VLP was determined at Paragon by quantitative Western blotting with antibody 6D8, using recombinant soluble GP protein with a C-terminal His-tag in place of the transmembrane peptide as the standard. Subsequent measurement of GP concentrations of VLP samples were done by ELISA at USAMRIID and gave results within 10% of those found by Western blotting. GP was typically 20–30% of the total protein as measured by bicinchoninic acid assay. To measure GP concentration by ELISA, a standard curve was prepared by adhering recombinant soluble GP protein overnight to an ELISA plate (Immulon 2HB from Thermo Fisher #3455) in carbonate buffer at pH 9.5 (Additional file 1: Figure S1). The soluble GP protein was expressed in HEK293 c18 cells, and had the transmembrane region replaced by a His-tag for purification. It was quantitated by UV absorbance (A_280_ 1 mg/mL = 1.30). GP on the ELISA plate was detected by probing with antibody 6D8 and an HRP-conjugated goat anti-mouse secondary antibody (Thermo Fisher #31430). The absorbance at 408 nm was fit to a 4-parameter logistic equation using SigmaPlot.

### Sonication and filtration

Sonication of VLP was done using a Branson Sonifier 250 with a 1/8 inch tapered microtip. To prepare VLP samples for further study, 0.5 mL of VLP in PBS were sonicated for 3 sets of 3 pulses of 1 s duration, at 50% duty cycle with the output control at 5.5. Samples were chilled on ice after each set of pulses.

Filtration was done by passage through a 2.5 cm Supor syringe filter (Pall) of either 0.45 µm or 0.8/0.2 µm pore size.

### Electron microscopy

Samples of VLP were adsorbed to formvar/carbon coated grids for electron microscopy and stained with PTA (phosphotungstic acid) or uranyl acetate. Samples were evaluated on a JEOL 1011 transmission electron microscope at 80 kV and digital images were acquired using an Advanced Microscopy Techniques (Danvers, MA, USA) digital camera system.

### Particle sizing

The particle size distribution of the samples was measured using an IZON qViro scanning ion occlusion sensing device. An IZON NP200A pore (nominal 200 nm diameter) was used, which was stretched until particles flowed freely. The voltage was 0.3–0.4 V and the pressure was varied up to 1.5 kPa. The sample introduced into the upper chamber contained 40 µL of VLP with 0.4–0.8 µg/mL [GP]. The buffer was PBS with 50 µg/mL Tween-80. Between 800 and 1,500 data points were collected for each experiment. The calibration standard was 217 nm polystyrene beads (SKP200B) diluted to 1 × 10^9^ particles/mL concentration.

Particle concentrations were determined from the pressure dependence of the event rate [17]. The concentration of an unknown sample (C_2_) was found from the slope of the pressure dependence of the sample’s event rate (g_2_), the calibration standard’s slope (g_1_), and the concentration of the standard (C_1_):

$$C_{2} = \left( {\frac{{g_{2} }}{{g_{1} }}} \right)C_{1}$$

### Conformational ELISA

VLP samples were diluted in 0.2 M sodium carbonate/bicarbonate buffer at pH 9.5 for coating of an Immulon 2HB plate (Thermo Fisher) overnight at 4°C. After washing four times with PBS/0.05% Tween-20, plates were blocked with 3% dry milk in PBS for 1.5 h at 37°C. The wells were probed with antibody 6D8, 6D3, or 13C6 in 02% casein/PBS. 6D8 was used at 2.5 µg/mL, 6D3 at 1 µg/mL, and 13C6 at 1.6 µg/mL. HRP-labeled goat anti-mouse-Fc (Thermo) was the secondary antibody. Plates were developed with an ABTS peroxidase substrate (KPL), stopped with 1% SDS, and the absorbance read at 408 nm.

### Nano-VLP purification

Transfection of HEK 293 c18 cells (ATCC CRL-10852) with expression vectors for Ebola Zaire GP and VP40 was done using PEI as transfectant and 500 mL shaker flask cultures. After 3 days growth, the culture was centrifuged to remove cells (1773×*g* for 15 min). The supernatant was then centrifuged at higher speed to pellet the VLP (18.6K×*g* for 2 h). The pellet from each liter of cells was resuspended in 10 mL of 10 mM sodium phosphate, 50 mM NaCl, pH 7.4 (buffer 1), and kept on ice. The resuspended pellet was sonicated to increase the fluidity of the sample and decrease the length of filamentous VLP, allowing for more efficient filtration.

Sonication of VLP was done using a Branson Sonifier 250 as described above. 10–12 mL of crude VLP in a 50 mL conical tube were sonicated for 4 sets of 3 pulses of 1 s duration, at 50% duty cycle with the output control at 5.5. Samples were chilled on ice after each set of pulses. After sonication, samples were diluted with an equal volume of buffer 1. The sample was then filtered once through a 2.5 cm GF/D filter (Whatman glass microfiber), and then twice through GF/F filters. These steps removed a quantity of yellow colloidal material. 20 mL of the filtered sample was then run over a Sartorius Sartobind S-75 membrane chromatography disc, pre-equilibrated with buffer 1 on a GE Healthcare FPLC system. The VLP flowed through the S-75 disc. Bound contaminants were eluted for analysis only using a gradient to 2 M NaCl. The flow rate was 1 mL/min at a backpressure of 0.16 MPa. S-75 discs were not reused.

The flow-through of the S-75 disc was again sonicated for 3 sets of 3 pulses to reduce particle size. The sample was then centrifuged in 50 mL conical tubes for 2 h at 14.6K×*g*) in a swinging bucket rotor. The pellet was resuspended in 2 mL of a sterile 0.5× PBS solution with 5% trehalose. The final filtration step to remove aggregates was done with a 2.5 cm Pall 0.45 µM Supor filter. Bioburden was tested by plating on LB agar at 37°C and no growth was found. Use of a 0.2 µM filter caused additional loss of the VLP and was not necessary to remove microbes for laboratory reagent purposes. Samples were then either stored frozen at −80°C, or lyophilized. Samples for SDS-PAGE were reduced and heated before loading on NuPage 4–12% Bis–Tris gels (Life Technologies).

### Lyophilization

For lyophilization, 262 µL volume samples in 0.5× PBS + 5% trehalose containing 60 µg GP each were placed in 2 mL glass vials. 5% trehalose was included in the buffer as a protectant against stresses associated with freezing and dehydration. The samples were frozen on dry ice, and then placed on the shelf of a VirTis AdVantage ES freeze-dryer at −20°C for drying in one stage. After 24 h exposure to vacuum (80 MT), the samples were reduced to a white powder. For stress-testing, the stoppered glass vial containing lyophilized VLP was placed in a 75°C block for 1 h. A 26G needle was inserted to vent the cap during heating. All samples were resuspended in sterile water at the time of use.

### In vivo efficacy

VLP were diluted in sterile saline for intramuscular administration. When the adjuvant poly-ICLC was used, it was diluted with the VLP in sterile saline and co-administered. Lyophilized VLP were reconstituted to approximately 500 µg/ml in sterile water prior to dilution in sterile saline. For all vaccination studies, female C57BL/6 mice (age 8–10 weeks) were vaccinated intramuscularly two times with 100 µl of volume, with 3 weeks between vaccinations. Two weeks after the final vaccination, peripheral blood was collected from vaccinated mice and antibody titers were measured using an IgG ELISA. Four weeks after the final vaccination, mice were challenged via the intraperitoneal (IP) route with 1,000 pfu of mouse adapted (ma)-EBOV [18]. Research was conducted under an IACUC approved protocol in compliance with the Animal Welfare Act, PHS Policy, and other Federal statutes and regulations relating to animals and experiments involving animals. The facility where this research was conducted, USAMRIID, is accredited by the Association for Assessment and Accreditation of Laboratory Animal Care International, and it adheres to principles stated in the 8th Edition of the Guide for the Care and Use of Laboratory Animals, National Research Council, 2011.

### Anti-glycoprotein ELISA

Anti-glycoprotein antibody titers were determined by ELISA. 2 µg/mL of recombinant Ebola virus glycoprotein was incubated in a flat bottom 96 well plate overnight. Glycoprotein with a C-terminal His-tag and lacking the transmembrane was expressed in HEK 293 c18 cells and purified by IMAC. Plates were incubated with blocking buffer (5% milk, 0.05% Tween in PBS) for 2 h, and then serum samples were added to plates. Samples were diluted by half log dilutions ranging from 2 to 5.5 logs. After 2 h, plates were washed with PBS + 0.05% Tween and goat anti-mouse IgG-HRP secondary antibody was added at a 0.6 µg/mL. One hour later, plates were washed and exposed using Sure Blue TMB 1-component substrate and stop solution (KPL), and the absorbance at 450 nm was recorded. Serum from unvaccinated animals was used to establish background and titers were defined as the serum dilution resulting in an absorbance greater than 0.2, where background was universally less than 0.2. Serum from animals previously determined to contain anti-glycoprotein antibody was included in each assay to serve as a positive control.

## Results

### Impact of sonication and filtration on the size distribution of VLP

**Figure 1:**
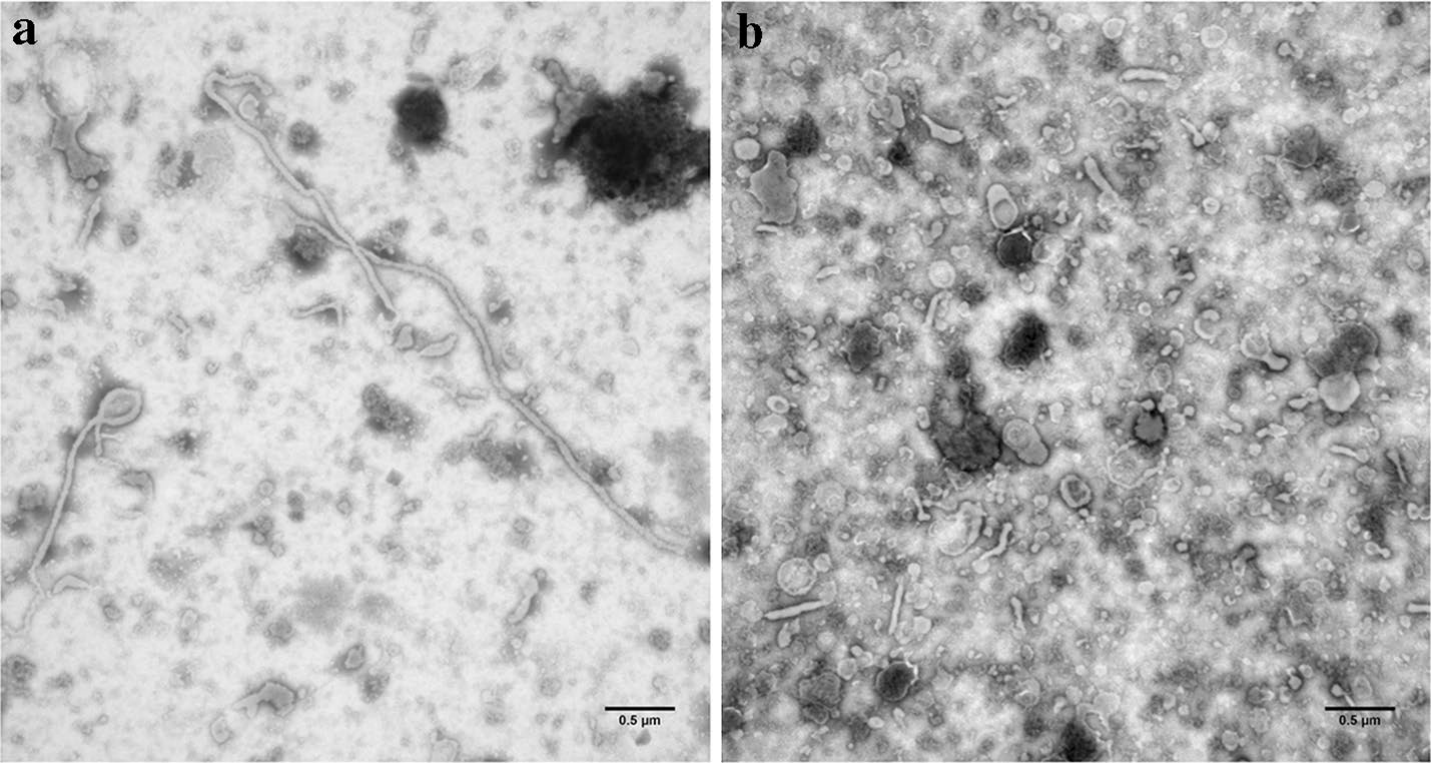
Electron micrographs of VLP before and after sonication. Bar is 0.5 µm. **a** Intact VLP. **b** Sonicated VLP.

As seen in earlier analyses [19], electron microscopy revealed that the Ebola Zaire VLP samples contained long filaments (>1 μm) with bulbous regions that resemble Ebola virions, spherical particles similar to the head region of the virus, and some large irregular objects that might be cellular debris (Figure 1a). Sonication of the VLP was optimized by varying the number of applied pulses until conditions were found that yielded fragments of up to ~400 nm length (Figure 1b) Sonication for a total of nine pulses was adequate to fragment the VLP, but more extensive disruption for a total of 30 pulses resulted in substantial loss of the integrity of the VLP membrane. Filtration of the sonicated samples through either a 0.45 µm or 0.8/0.2 µm cutoff syringe filter yielded both spherical particles and fragments of filaments, and effectively removed the larger particles and aggregates. Sonication of VLP improved the yield of GP through a 0.8/0.2 μm filter from 10 to 50%, and through a 0.45 μm filter from 28 to 63%.

The size distribution of the VLP samples was analyzed by Scanning Ion Occlusion Sensing (SIOS). In this method, particles in the sample are forced through a pore of adjustable size by an applied pressure and an electroosmotic potential. As particles pass through the pore, a change in current due to pore blockage is measured as a function of time. The amplitude of the decrease in current is proportional to the degree to which the pore is blocked, while the duration of the blockade event is the time required for the particle to transit the pore. One advantage of this method lies in the characterization of heterogeneous samples containing particles of different sizes and shapes, which may be detected as individual blockade events rather than averaged over the whole population.

**Figure 2:**
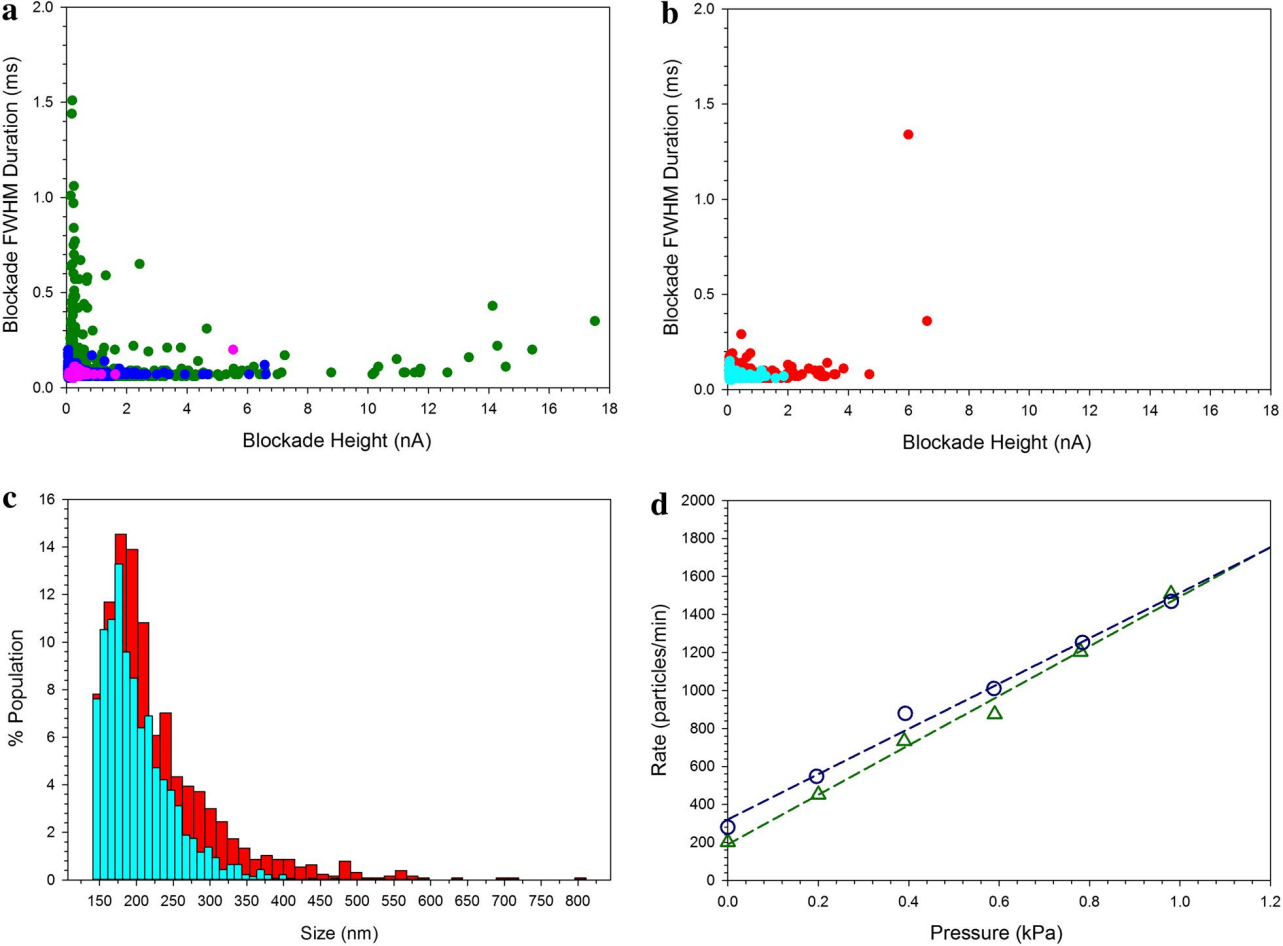
Nanopore measurements. **a** Intact VLP (*green*) sonicated VLP (*blue*) and 217 nm polystyrene beads (*pink*). **b** VLP after sonication and passage through a 0.45 µm (*red*) or 0.8/0.2 µm (*cyan*) filter. **c** The calculated particle size distribution of filtered VLP samples after calibration using the polystyrene beads. **d** Pressure dependence of the particle flow rate. Calibration beads (*green triangles*), sonicated and filtered (0.45 μm) VLP (*blue circles*). R^2^ was 0.99 for both linear regressions.

SIOS analysis of the undisrupted VLP yielded a bifurcated plot of the blockade event duration in ms (FWHM: full width at half maximum) vs. blockade height in nA (Figure 2a). The events of lesser height and longer duration in the VLP sample are consistent with the transit of narrow, elongated filamentous particles through the pore. VLP filaments, which are 70–85 nm wide and up to 4 µm in length, may be expected to block the pore only partially, while taking a relatively long period of time to transit. In comparison, the spherical 217 nm polystyrene beads used for calibration showed a tight cluster of blockade events of less than 2 nA magnitude and 0.2 ms duration. Disruption of the VLP by sonication (Table 1) resulted in the loss of most events with greater blockade amplitudes (>2 nA) and nearly all those of longer duration (>0.2 ms), consistent with the disruption of filaments observed in the EM. Passage of the sonicated VLP through a 0.8/0.2 μm syringe filter removed the larger remaining particles (Figure 2b).

**Table 1:** Nanopore analysis of VLP

Sample	% FWHM >0.2 ms	% Height >2 nA	Mean particle size (nm)
Undisrupted	6.7	6.2	
Sonicated	0.1	1.9	227
Sonicated and filtered 0.45 µm	0.3	2.1	230
Sonicated and filtered 0.8/0.2 µm	0	0	202
Incubated 37°C 24 h	2.7	11	
Incubated 37°C 96 h	2.7	9.1	

Figure 2c shows the calculated particle size distributions of the filtered samples, which were consistent with what was observed by EM. The mean particle size is normally calculated from SIOS data by calibration using beads of known size [20]. However, this analysis may not be appropriate when the sample contains a significant fraction of elongated, non-spherical particles. We therefore present mean particle sizes only for the sonicated samples (Table 1), which have a relatively narrow distribution of particle sizes and shapes. The smaller particles of less than 150 nm could not be simultaneously resolved in this nanopore measurement; however we have chosen to focus the particles >150 nm that were identifiable as VLP or fragments of VLP.

SIOS can also be used to determine particle concentrations from the rate of observed events compared to a calibration standard of known concentration [17]. However, passage of intact VLP samples through the SIOS pore was frequently interrupted by blockages, presumably due to the larger aggregates present. These blockages did not affect the particle size measurements, which were determined from individual events. Experiments could be resumed by pipetting the sample up and down, but a consistent flow rate of particles through the pore could not be obtained. By removal of larger particles or aggregates from the sample, filtration allowed a smooth flow through the pore for more than 1 min.

Measurement of the rate of events at various applied pressures (Figure 2d) was then done to allow calculation of the particle concentration from the relative slopes of the pressure dependence curves. For the filtered VLP sample shown, the concentration was 8.2 ± 0.8 × 10^11^ particles/mL (±SE, n = 3).

### Thermostability of GP in VLP

GP only comprises approximately 20–30% of the total protein in VLP preparations, but it is the most significant component in terms of immunogenicity [6]. The conformational integrity of GP is hypothesized to be important for immunogenicity. We therefore sought to develop an ELISA method that would characterize specifically the conformation of GP in VLP preparations subjected to applied stress. In this approach, we compared the relative amount of binding of antibodies that recognize either linear or conformational epitopes. Denaturation of GP would be expected to result in loss of recognition by the conformationally-sensitive antibodies, while binding of the linear epitope antibody would be maintained.

Antibody 6D8 recognizes a linear epitope of GP (a.a. 389–405), while 13C6 and 6D3 are conformationally-dependent [21, 22]. 13C6 is known to bind to the glycan cap [23]. All are non-competing. We anticipated that if GP were denatured by applied stress, binding by 6D8 should remain relatively constant, while binding by 6D3 and 13C6 should decrease.

**Figure 3:**
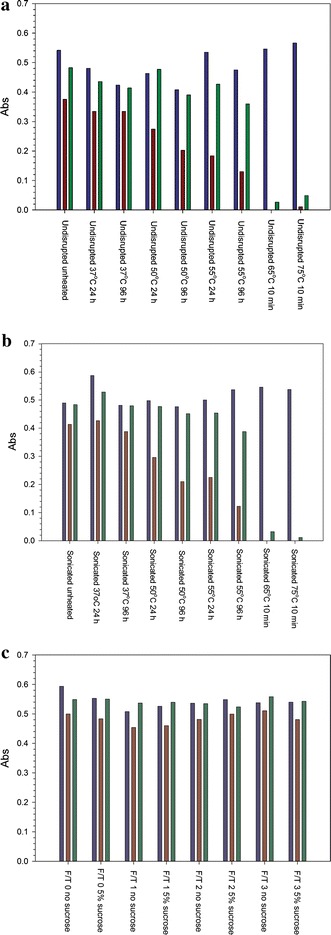
Accelerated degradation of VLP. **a** Undisrupted VLP incubated at elevated temperatures for the times indicated and probed by antibodies 6D8 (*blue*), 6D3 (*red*), or 13C6 (*green*). The absorbance at 408 nm is shown on the y-axis. **b** Sonicated VLP, same as part A. **c** Undisrupted VLP were subjected to repeated rounds of freeze/thawing in the presence or absence of 5% sucrose.

We used the ELISA method to test the resistance of both untreated, filamentous VLP and sonicated VLP to accelerated degradation by thermal stress. VLP samples were incubated at elevated temperatures for the indicated time periods in Figure 3a, b. Under all conditions tested, there was no difference between the impacts of thermal stress on sonicated VLP as compared to intact VLP, indicating that fragmentation of the VLP did not affect the conformational stability of GP.

Incubation at 37°C for up to 96 h had no significant effect on the conformational integrity of GP, as judged by binding of the conformationally sensitive 6D3 and 13C6 antibodies. Binding of the control 6D8 was unchanged by incubation at the higher temperatures. At 50°C or 55°C for 24 or 96 h, partial loss of 6D3 binding was observed, and after only 10 min at 65 or 75°C, 13C6 binding was also lost almost completely. The reason for the more rapid loss of 6D3 binding on heating is not yet clear, but could be due to partial denaturation of domains of the GP as temperature increased.

While incubation at 37°C for as long as 96 h did not result in denaturation of GP, the integrity of filaments was affected. After incubating a VLP sample for 24 h at 37°C, we observed a decreased population of particles with long-duration pore transits (Table 1 and Additional file 1: Figure S2A). This result indicates that longer filaments are not stable to extended incubation at 37°C, although GP conformation could be maintained even when filamentous structure was disrupted.

Having determined the sensitivity of GP in VLP to heating, we next evaluated the effects of repeated freeze/thawing on VLP particle size and the GP conformation. The VLP preparation used for these studies was purified at USAMRIID and tested after having never been frozen, or having been subjected to 1–3 rounds of freeze/thawing in PBS or PBS + 5% sucrose. VLP were thawed in a 37°C water bath and refrozen on dry ice. No significant change in GP conformation (Figure 3c) as a result of freeze/thawing was found. Electron microscopy and particle sizing measurements also found no significant change in size as a result of freeze/thawing (Additional file 1: Figure S2B).

### Immunopotency testing

Based on our biochemical assessments, we had shown that optimized sonication can result in smaller, more homogenous VLP that expressed conformationally intact GP, while excessive heating of the VLP disrupted the GP conformation. To evaluate the relevance of these findings in terms of vaccine efficacy, we vaccinated C57BL/6 mice with VLP treated under the respective conditions and challenged them with ma-EBOV. Based on previous studies, we had shown that 10 µg of VLP, based on GP content, was the minimum sufficient vaccine dose to reliably achieve 90–100% protection from challenge [24]. We therefore used this dose level as our baseline for analysis.

**Figure 4:**
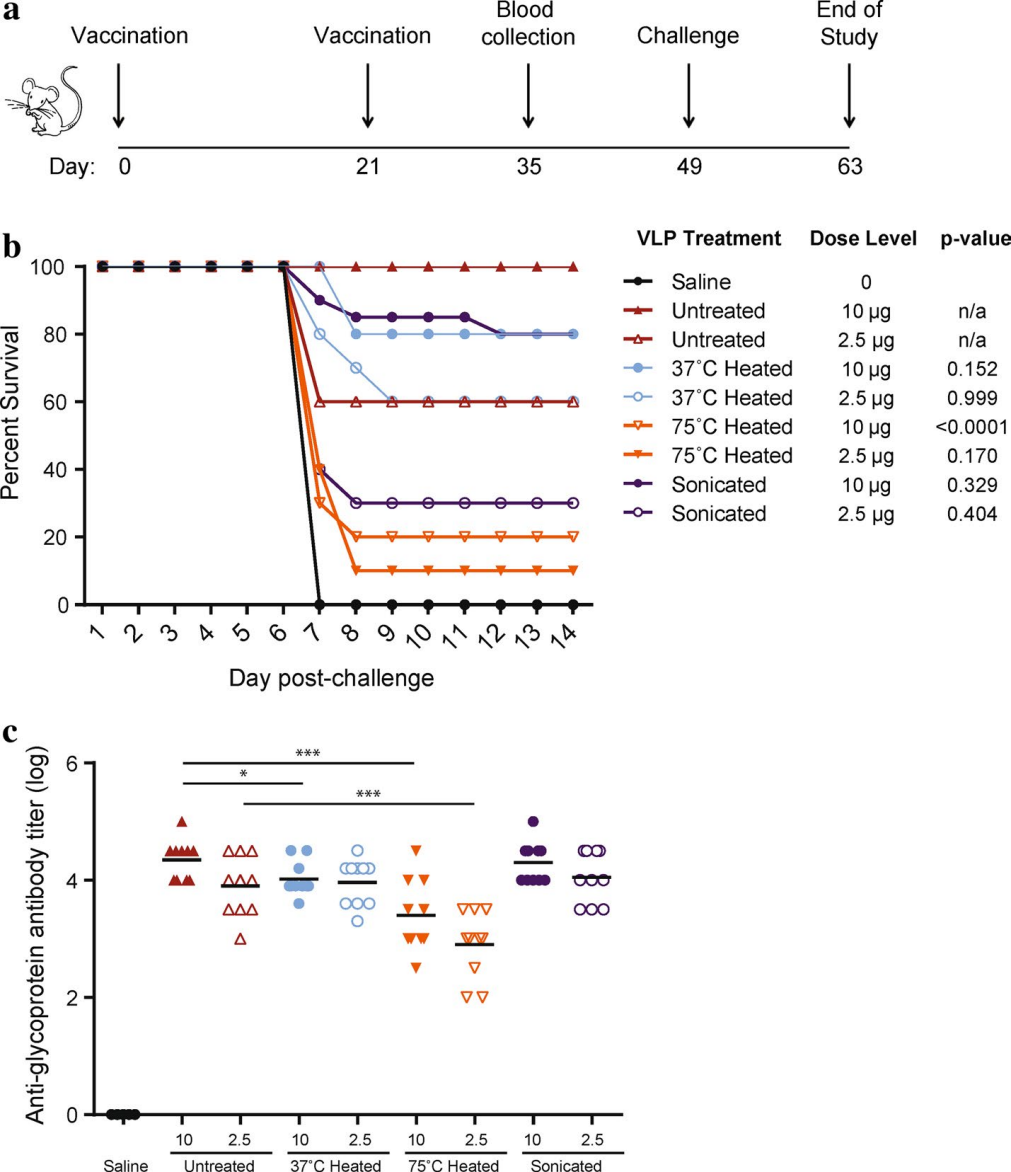
Impact of heating or sonication on VLP immunogenicity. **a** Schematic depicting vaccination schedule. **b** C57BL/6 mice were vaccinated two times, with 3 weeks between vaccinations. VLP were untreated, heated at 37°C for 96 h, heated at 75°C for 15 min, or sonicated. Mice were challenged 4 weeks after the final vaccination and the percent survival is shown. *p*-values determined using Fisher’s exact test to compare survival of each treatment group at the given dose level to control VLP (“untreated”). **c** Anti-glycoprotein antibody titers of vaccinated mice measured from serum collected 2 weeks after the final vaccination. VLP dose level (10 or 2.5 µg) and treatment (untreated, heating conditions, and sonication status) are indicated on the x-axis. P values determined using one-tailed Student’s *t* test where *p < 0.05, **p < 0.005, ***p < 0.0005.

To evaluate the impacts of heating and sonication on VLP efficacy, we vaccinated animals with either 10 µg or 2.5 µg of VLP two times, on the schedule shown in Figure 4a. As shown in Figure 4 and Additional file 1: Table S1 (experiment #1), VLP that were sonicated or heated at 37°C for 96 h provided comparable protection to filamentous control VLP that were not treated, suggesting that these conditions did not impact immunogenicity. In contrast, VLP that were heated at 75°C for 15 min did not provide protection from challenge, supporting a relationship between GP conformational integrity and immunogenicity. The *p*-values comparing survival of groups treated with control VLP or 75°C heated VLP confirmed the deleterious effect of high temperature, while the effects of sonication or 37°C incubation were not significant. Anti-GP antibody titers in animals vaccinated with VLP heated at 75°C were also significantly lower than the titers achieved in animals vaccinated with undisrupted VLP, sonicated VLP, or VLP heated at 37°C, at both dose levels tested (Figure 4).

**Figure 5:**
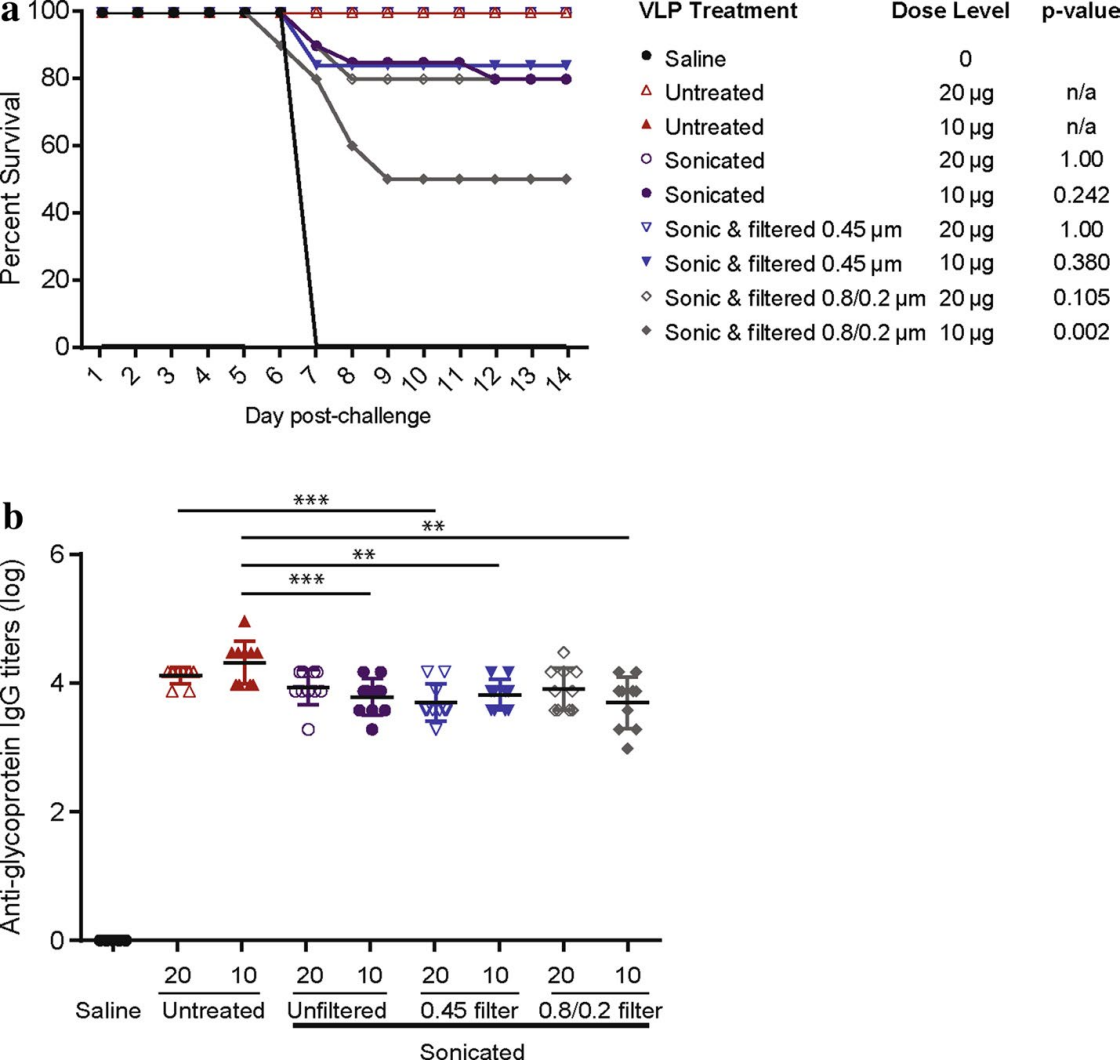
Impacts of sonication and filtration on VLP immunogenicity. **a** C57BL/6 mice were vaccinated two times, with 3 weeks between vaccinations, as in Figure 4a. VLP were untreated, sonicated, or sonicated and passed through either a 0.45 µm or 0.8/0.2 µm filter (indicated on x-axis). The percent survival is shown. *p*-values determined using Fisher’s exact test to compare survival of each treatment group at the given dose level to control VLP (“untreated”). **b** Anti-glycoprotein antibody titers of vaccinated mice measured from serum collected 2 weeks after the final vaccination. VLP dose level (10 or 20 µg) is indicated on the x-axis. P values determined using one-tailed Student’s *t* test where *p < 0.05, **p < 0.005, ***p < 0.0005.

Considering that we had not observed 100% protection with the 10 µg dose level of sonicated VLP, we chose to vaccinate animals with 10 or 20 µg of VLP when comparing the impact of filtration on protection (Figure 5 and Additional file 1: Table S1, experiment #2). Sonicated VLP that were filtered through a 0.45 µm filter displayed comparable immunogenicity to those that were not filtered and to undisrupted VLP. VLP that were sonicated and then filtered with a 0.8/0.2 μm cutoff had slightly lower efficacy, which was statistically significant (*p* value <0.05) at the 10 µg dose, though antibody titers were comparable between all groups.

### Nano-VLP

The results above suggested to us that difficulties in manufacture of full-length filamentous VLP might be overcome by a reduction in their size, without loss of potency. We then developed a procedure for purification of nano-VLP consisting of a combination of centrifugation, sonication, glass fiber filtration, and Sartobind S-75 membrane negative capture steps; with a final filtration for removal of aggregates and bioburden. The SDS-gel electrophoresis pattern of the nano-VLP product and a preparation made by the sucrose-gradient procedure [3] are shown in Additional file 1: Figure S3. The protein bands appear very similar in migration and relative amounts. We also verified that the nano-VLP preparation possessed appropriate GP conformation by ELISA as done above (not shown). Measurement of the GP yield by ELISA with antibody 6D8 gave figures of 1.6–2.0 mg/L of cell culture, in comparison with ~1 mg/mL for the sucrose gradient method. Both contained 20–30% GP when total protein was measured by BCA. A final 0.45 μm filtration provided adequate bioburden removal for laboratory usage, yielding a product that showed no bacterial growth on plating. Filtration with a 0.8/0.2 μm filter was possible but resulted in additional loss of product.

**Figure 6:**
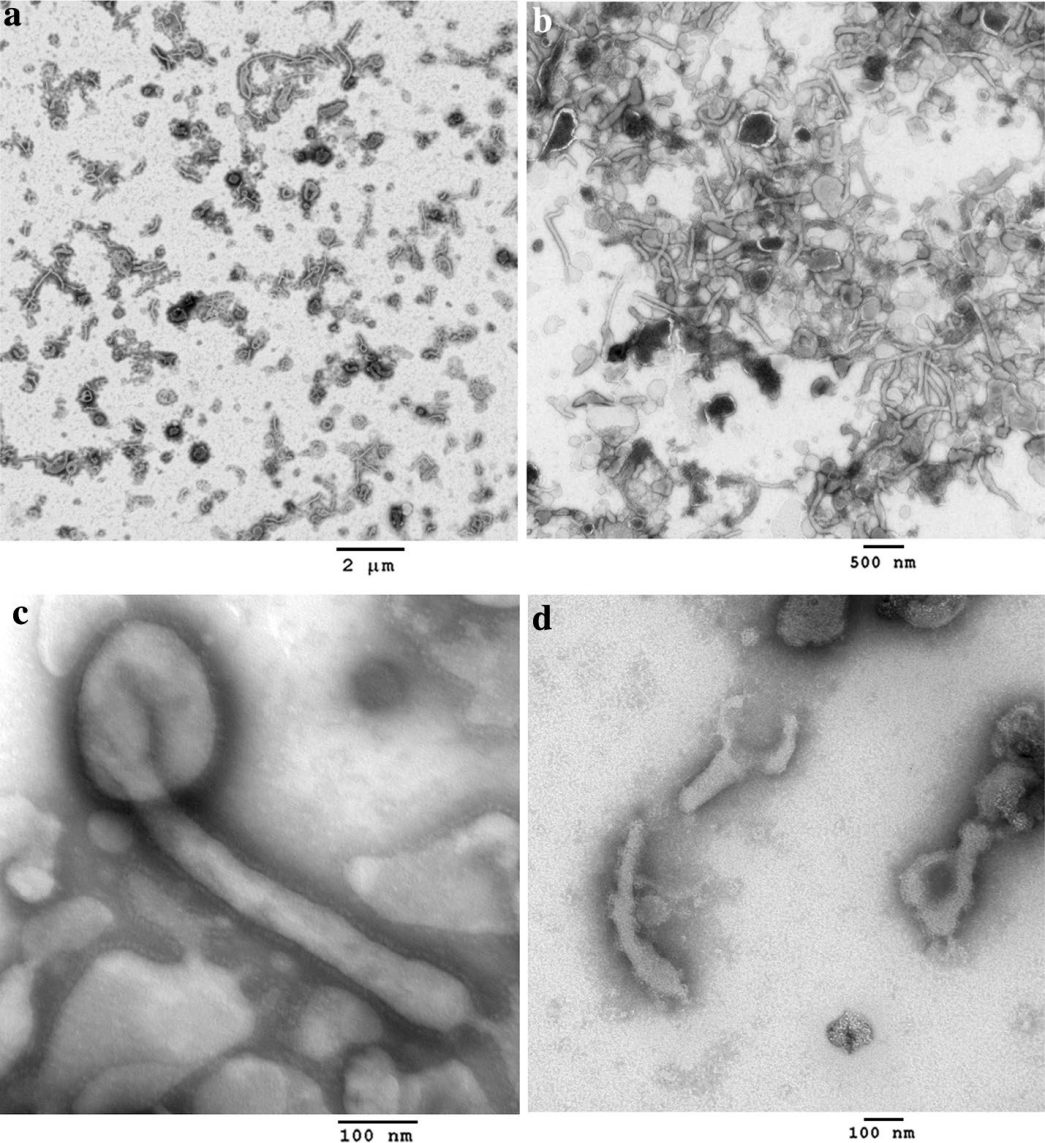
Electron micrographs.** a** Nano-VLP. *Bar* 2 µm. **b** Lyophilized nano-VLP, after resuspension. *Bar* 0.5 µm. **c** Lyophilized nano-VLP. *Bar* 0.1 µm. **d** Lyophilized nano-VLP after heating to 75°C for 1 h. *Bar* 0.1 µm.

**Figure 7:**
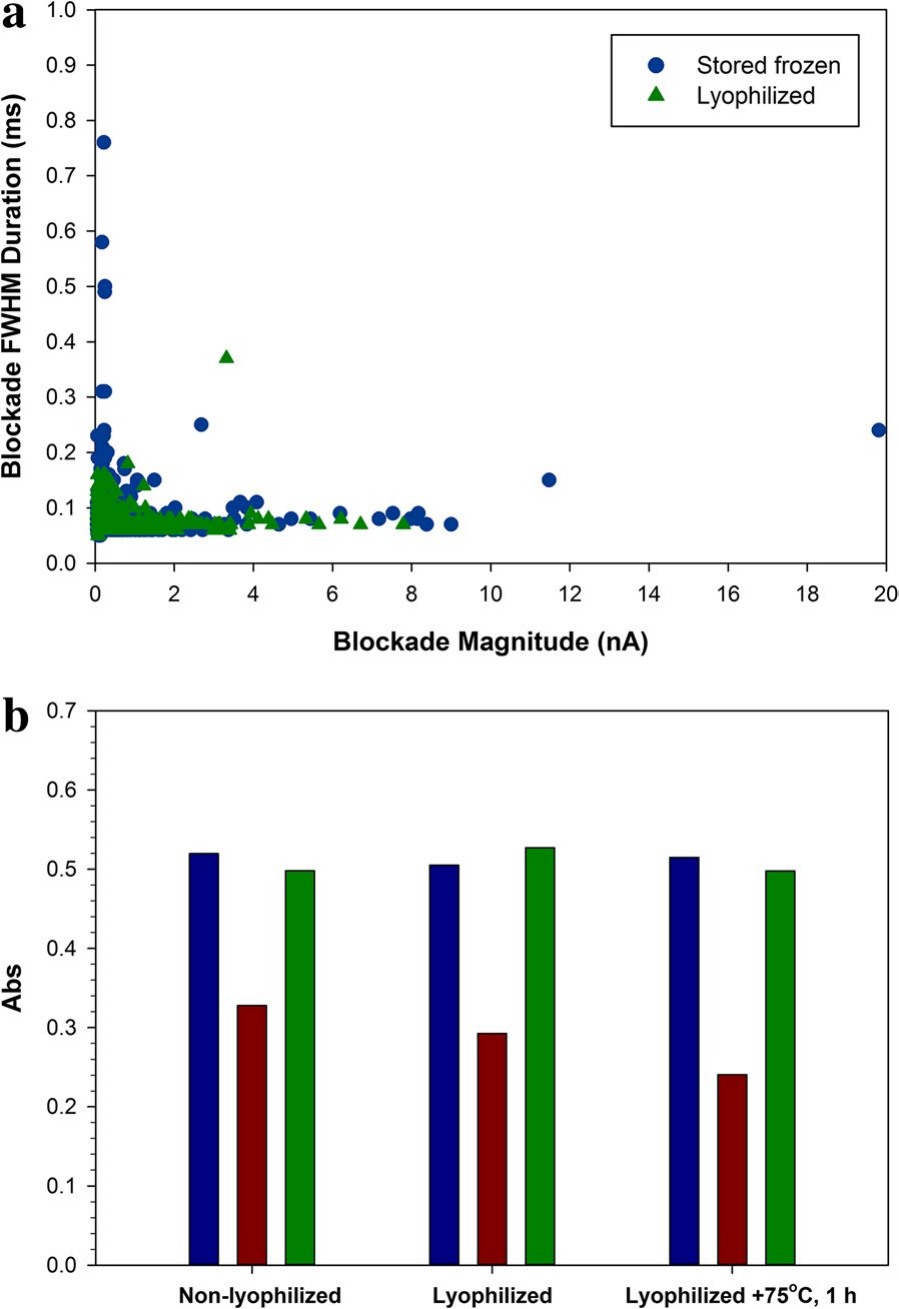
**a** Nanopore event duration (ordinate) and blockade magnitude (abscissa) of non-lyophilized (*blue circles*) and lyophilized (*green triangles*) nano-VLP. **b** ELISA to probe the conformational integrity of GP in nano-VLP that were not lyophilized, lyophilized, and lyophilized and heated to 75°C for 1 h. *Blue bars* linear epitope antibody 6D8, *red bars* conformational antibody 6D3, *green bars* conformational antibody 13C6.

Transmission electron microscopy of the nano-VLP with PTA staining showed linear, branched, spherical and “6” shaped particles (Figure 6a). Linear filaments were as long as 1.5 microns, but most particles were shorter filaments or spheres of ~200 nm diameter. Particle size was also examined using a qViro device (Figure 7a). With the nano-VLP sample, 7.1% of observed events had a passage duration of >0.2 ms. These events may represent passage of the shorter filaments found in Figure 6a. Results from the qViro nanopore analysis appeared to be consistent with the EM data. The nano-VLP were not observed to possess lengths of several microns as is commonly found in sucrose-gradient purified VLP and authentic virions. The mean particle diameter was calculated to be 230 nm using a spherical bead standard; however, this method does not take into account the shape of the filament fragments. Although some of the fragments of filaments present were longer than the 0.45 micron pore size filter used in this preparation, their width was less than 100 nm.

We tested lyophilization of the nano-VLP as a possible means to formulate for stable storage. Figure 6b, c show electron micrographs of the lyophilized nano-VLP, after resuspension in water. The filaments retained structure after lyophilization, although some shortening of filaments occurred, which was confirmed by nanopore analysis (Figure 7a). The calculated mean particle diameter of the lyophilized nano-VLP was 217 nm. The GP layer coating the lyophilized nano-VLP was visualized by negative staining in Figure 6c, and was detected by immunogold-staining with antibody 6D8 (Figure S4). Retention of the folded conformation of GP in the nano-VLP after lyophilization was confirmed using ELISA (Figure 7b). Lyophilized VLP resuspended readily without the appearance of aggregation, and the particles flowed easily through the nanopore.

As described above, we observed that the GP of Ebola VLP underwent denaturation when heated in liquid suspension to 75°C for 15 min, which was correlated with a nearly complete loss of protective capability in the mouse model. However, we found that lyophilized nano-VLP could be heated in the dry form before resuspension to 75°C for at least 1 h, with little apparent loss of GP conformation as determined by conformational ELISA (Figure 7b). Electron micrographs of the heated, lyophilized nano-VLP (Figure 6d) showed that the filament fragments were relatively unchanged, although increased irregularities on the membrane surface were apparent.

### Efficacy as a vaccine

**Figure 8:**
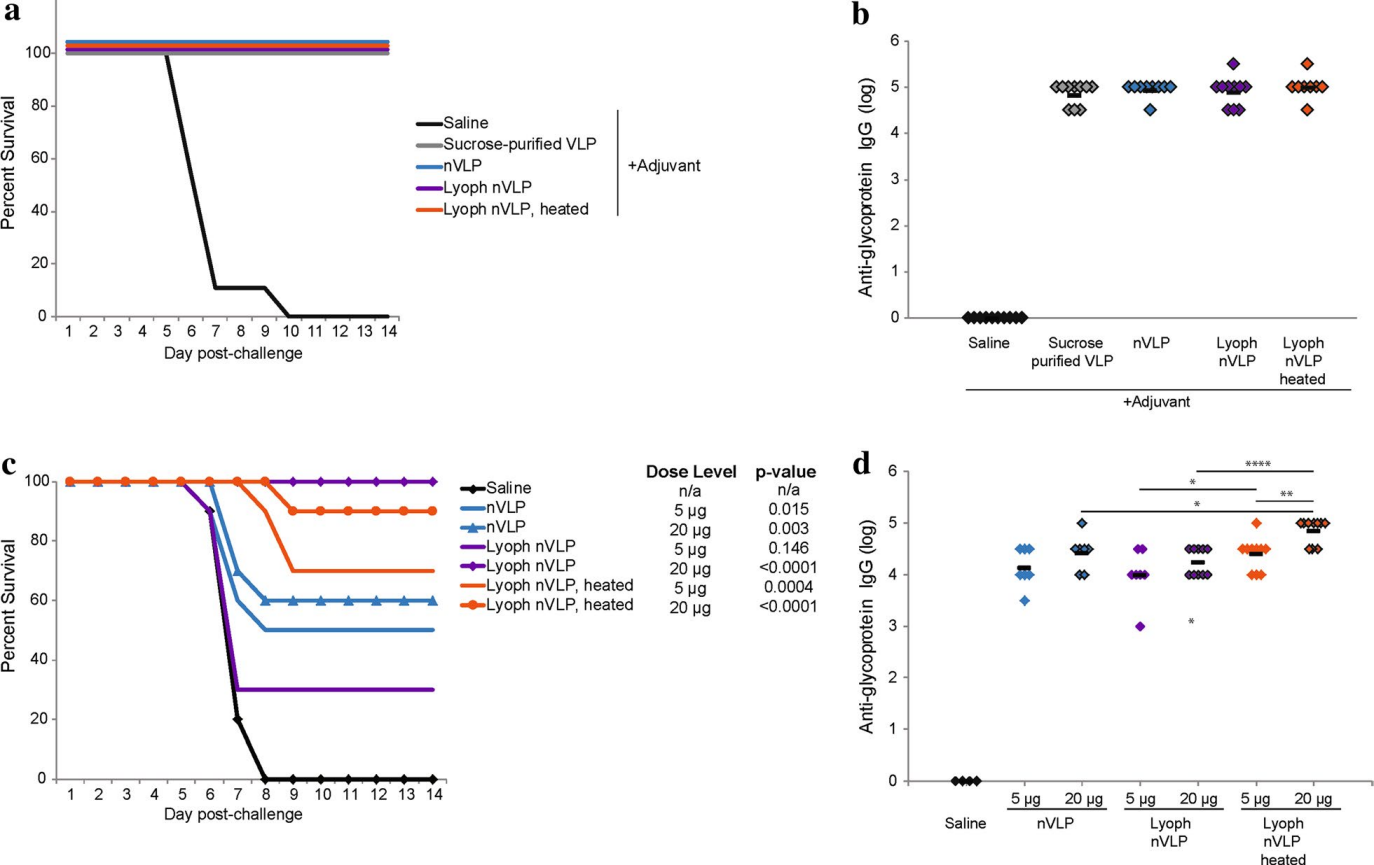
**a** C57BL/6 mice were vaccinated two times, with 3 weeks between vaccinations, as in Figure 4a. Dose level of VLP or nano-VLP (nVLP) was 5 µg, based on GP content, and dose level of the adjuvant poly-ICLC was 10 µg. Time to death in days, expressed as percentage survival in each group. All animals except those in the adjuvant-only poly-ICLC control group survived. **b** Anti-glycoprotein titers of mice from A inoculated with adjuvant alone, or adjuvant plus sucrose-gradient control VLP, frozen nVLP, lyophilized nVLP, and lyophilized nVLP heated to 75°C for 1 h. **c** Time to death in days, expressed as percentage survival in each group of mice vaccinated with 5 or 20 µg nVLP doses, without adjuvant. The experiment compares results for nVLP stored frozen, lyophilized nVLP, lyophilized and heated nVLP, and a control of sucrose-gradient purified VLP. *p*-values determined using Fisher’s exact test to compare survival of each treatment group vs. saline. **d** Anti-glycoprotein titers of mice from **c**. P values determined using one-tailed Student’s *t*-test where *p < 0.05, **p < 0.005, ***p < 0.0005. “Lyoph” indicates lyophilized material.

The ability of the nano-VLP to confer protection from mouse-adapted Ebola challenge was tested in a mouse assay. Figure 8a shows results of an experiment in which mice were vaccinated with 5 µg doses of various VLP preparations [GP content of VLP] in combination with 10 µg poly-ICLC adjuvant. The VLP preparations that were tested are as follow: sucrose-gradient purified VLP, which served as a bridging control to previous studies; nano-VLP that were stored frozen; nano-VLP that were lyophilized; and lyophilized nano-VLP that had been heated in the vial at 75°C for 1 h prior to resuspension. All (10/10) animals vaccinated with adjuvant only died after challenge with mouse-adapted Ebola virus, while all in the VLP-vaccinated groups survived (Figure 8a). The anti-GP titers in the VLP-containing groups were uniformly high (Figure 8b).

As a more stringent test of immunogenicity, we performed an experiment without the use of adjuvant (Figure 8c, d). nano-VLP were administered at doses of 5 or 20 µg GP content (Additional file 1: Table S2). For each group, 20 µg conferred better protection than 5 µg. At the 20 µg dosage, nano-VLP that had been stored frozen yielded 6/10 survival, while lyophilized nano-VLP gave 10/10 survival and lyophilized/heated VLP gave 9/10. For all of these groups except lyophilized nano-VLP at 5 µg, survival vs. saline control was statistically significant. However, differences in survival between the vaccine groups, including the filamentous VLP used in earlier studies, were not statistically significant. Anti-GP titers also showed a dose response, and titers for the heated, lyophilized VLP were significantly higher than titers for lyophilized nano-VLP or frozen nano-VLP at the 20 µg dose level. The reason for this higher titer is not known.

## Discussion

### VLP stability and GP conformation

Vaccines that can withstand ambient temperatures for extended periods are highly desirable to avoid loss of potency if the cold chain is broken. Thermal stability studies on VLP indicated that the GP antigen can withstand moderately high temperature stress for an extended period. Incubation at 55°C for more than 24 h resulted in partial loss of conformation, while at 75°C denaturation occurred rapidly as measured by ELISA. No significant loss of GP conformation was observed with 96 h incubation at 37°C, however. Loss of conformational integrity in the ELISA was correlated to loss of immunopotency in a mouse bioassay, demonstrating that the conformational ELISA method introduced herein can be used to predict rapidly the decreased potency of thermally stressed Ebola VLP vaccine samples. Structural studies of neutralizing antibodies to Ebola GP support the importance of GP conformation in antibody recognition [25].

Only one other study has examined filovirus VLP stability systematically, to our knowledge [26]. Those authors used biophysical methods on VLP derived from a baculovirus expression system, while we employed a human cell-based system and developed approaches to focus specifically on antigenic integrity. The light scattering and circular dichroism measurements of Hu et al. indicated that at neutral pH Ebola VLP’s lost conformational integrity above 50–60°C. Although the thermal transitions were broad, and global properties of the VLP were assayed rather than those of GP specifically, those results are consistent with ours.

### Particle size vs. immunogenicity

Previous studies have found that particles greater 500 nm in size are processed more slowly by the immune system due to exclusion from direct drainage to lymph nodes [11]. This fact might suggest that large filamentous VLP would be less immunogenic than smaller VLP. However, we found no significant difference in potency between the filamentous and sonicated VLP using the mouse vaccination model. The roughly 230 nm average size of the sonicated VLP was large enough to provide the repeated antigen array necessary for strong stimulation of the immune response by GP [10]. Passage of the sonicated VLP through a 0.45 µm filter did not appear to affect potency, but passage through a 0.8/0.2 µm filter resulted in a lower survival rate after challenge. However, a statistically significant difference in potency of the more stringently filtered VLP was observed only at the 10 µg dose (Additional file 1: Table S1).

To the extent that the mouse model is predictive, our results indicate that the presence of intact filaments is not essential to a VLP vaccine. The finding that reduction of particle size by sonication did not greatly change immunogenicity may be due at least in part to disruption of longer VLP filaments in vivo. We observed that extended incubation at 37°C resulted in shortening of filaments, as determined by nanopore sizing measurements. This suggests that VLP filaments will breakdown into pieces after injection into the body of an animal, with the result being that the large initial size of the VLP may not be an impediment to access to the lymphatic system.

### Nano-VLP and progress in vaccine development

We have described a new process for purification of a filovirus VLP vaccine that is more rapid and easily scaled for manufacturing than the previous sucrose gradient-based method. The size of the nano-VLP was smaller than that of complete Ebola virions, with spherical particles of roughly 230 nm diameter, and filaments of around 500 nm length. Nevertheless, the Ebola nano-VLP remained larger than typical viruses and GP in the native conformation was present on the surface of the nano-VLP. Reduction in size of the VLP facilitated their purification by membrane chromatography, which is more easily adapted to large-scale production than a sucrose gradient method. The resulting product was also more uniform in size, although differences in morphology between spherical and filamentous fragments remained. Further advancements will be necessary to bring our laboratory-scale method to cGMP scale and meet all FDA criteria for product licensure.

We have also presented assays for in vitro testing of GP conformational integrity in the context of VLP, the presence and relative population of filaments, and the concentration of particles in filtered VLP samples. The observed correlation of the conformational ELISA with the mouse immunopotency assay may allow its use in the future as a more rapid quality-control assay for the VLP and other types of Ebola vaccines, while the concentration of particles can be measured using the rate of events observed during flow through a nanopore.

Our results indicated that the lyophilized nano-VLP preparation was highly thermostable, but further studies will be needed to determine if long-term storage of the lyophilized formulation without refrigeration is possible. Elimination of a cold chain requirement would decrease costs associated with the vaccine and greatly ease distribution to locales without reliable electricity, especially important for a tropical disease [27].

## Conclusions

We developed a procedure to reduce the size of filovirus VLP, which permitted us to purify “nano-VLP” using chromatography and filtration. We found that Ebola VLP can be reduced to a more uniform and easily manipulable size range of roughly 230 nm diameter, while retaining GP conformational integrity and the antigenic effectiveness of the vaccine. Additionally, the nano-VLP can be lyophilized without loss of GP and VLP structure and immunogenicity. Lyophilized nano-VLP have greatly enhanced thermostability, suggesting that the creation of an Ebola VLP vaccine without a cold chain requirement is possible.

## Additional file

**Additional file 1: Figure S1.** Quantitation of GP in VLP by ELISA. **Figure S2.** Nanopore measurements of VLP samples. **Figure S3.** SDS-PAGE comparison of Nano-VLP preparation with sucrose-gradient purified VLP. **Figure S4.** Electron micrographs of Nano-VLP with immunogold staining. **Table S1.** Survival of vaccinated mice after ma-Ebola challenge. **Table S2.** Survival of mice vaccinated with Nano-VLP without adjuvant.

## Abbreviations

VLP: virus-like particles
GP: glycoprotein
SIOS: scanning ion occlusion sensing
PTA: phosphotungstic acid
PBS: phosphate-buffered saline
