# Supplementary material for: A thermostable, chromatographically purified Ebola nano-VLP vaccine
Source: J Transl Med. 2015 Jul 15;13:228. doi: 10.1186/s12967-015-0593-y (PMC4502941; doi:10.1186/s12967-015-0593-y)
Supplement: Supplementary file 1 — Additional file 1: Figure S1. Quantitation of GP in VLP by ELISA. Figure S2. Nanopore measurements of VLP samples. Figure S3. SDS-PAGE comparison of Nano-VLP preparation with sucrose-gradient purified VLP. Figure S4. Electron micrographs of Nano-VLP with immunogold staining. Table S1. Survival of vaccinated mice after ma-Ebola challenge. Table S2. Survival of mice vaccinated with Nano-VLP without adjuvant. [file 12967_2015_593_MOESM1_ESM.pdf]

Supplemental Data

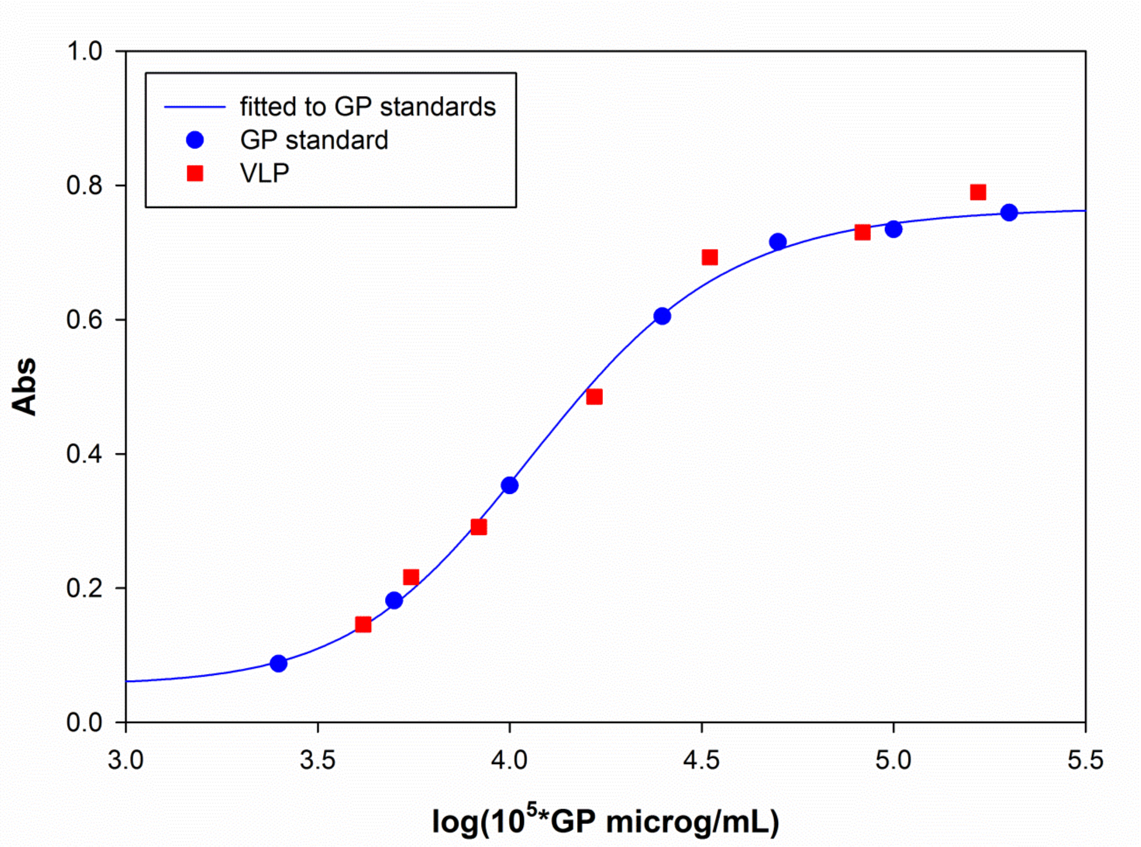

Figure S1. Quantitation of GP in VLP by ELISA. The y-axis is the absorbance at 408 nm. Blue circles are the GP standard data, and the blue line was fitted using a 4-parameter logistic equation with SigmaPlot. Red squares are data obtained from VLP, plotted assuming the concentration of 830 µg/mL [GP] previously derived by quantitative Western blotting. The close agreement of the VLP data points with the standard curve indicated that ELISA method and Western blot gave similar results. The [GP] concentration calculated by fitting the third VLP ELISA data point to the standard curve was 805 µg/mL.

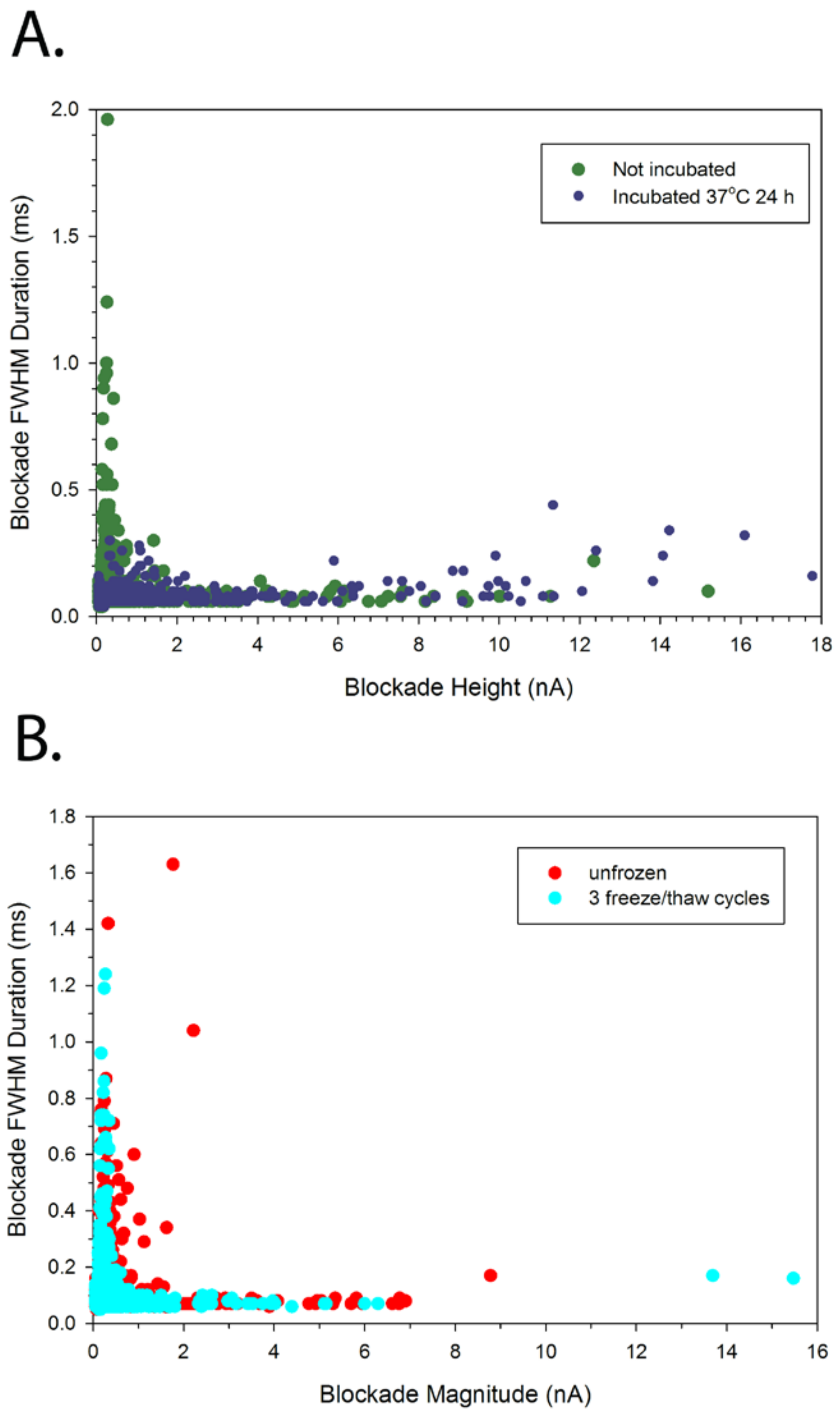

Figure S2. A. Nanopore measurements of VLP samples either incubated at 37ºC for 24 h (blue), or not incubated (green). B. VLP samples unfrozen (red) or after 3 freeze/thaw cycles (cyan).

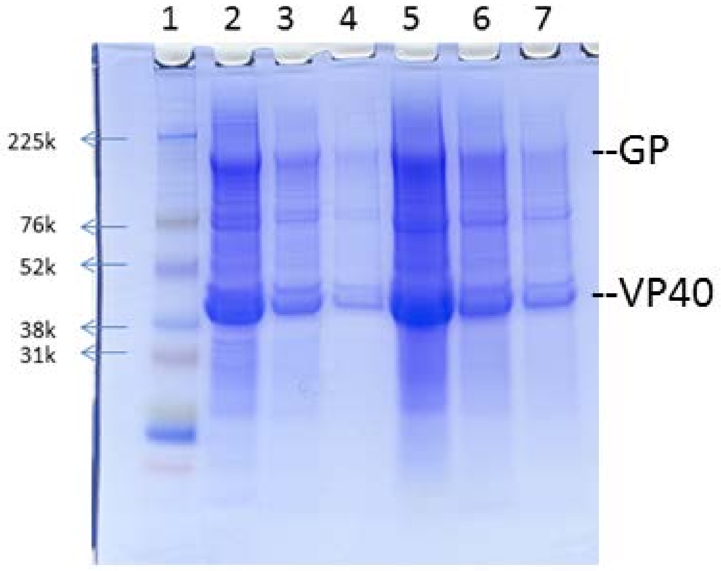

Figure S3. SDS-PAGE comparison of Nano-VLP preparation with sucrose-gradient purified VLP. Lane 1- MW marker. 2- 15 µL of Nano-VLP. 3- 5 µL of Nano-VLP. 4- 2 µL of Nano-VLP. 5- 15 µL of sucrose-gradient VLP. 6- 5 µL of sucrose-gradient VLP. 7- 2 µL of sucrose-gradient VLP.

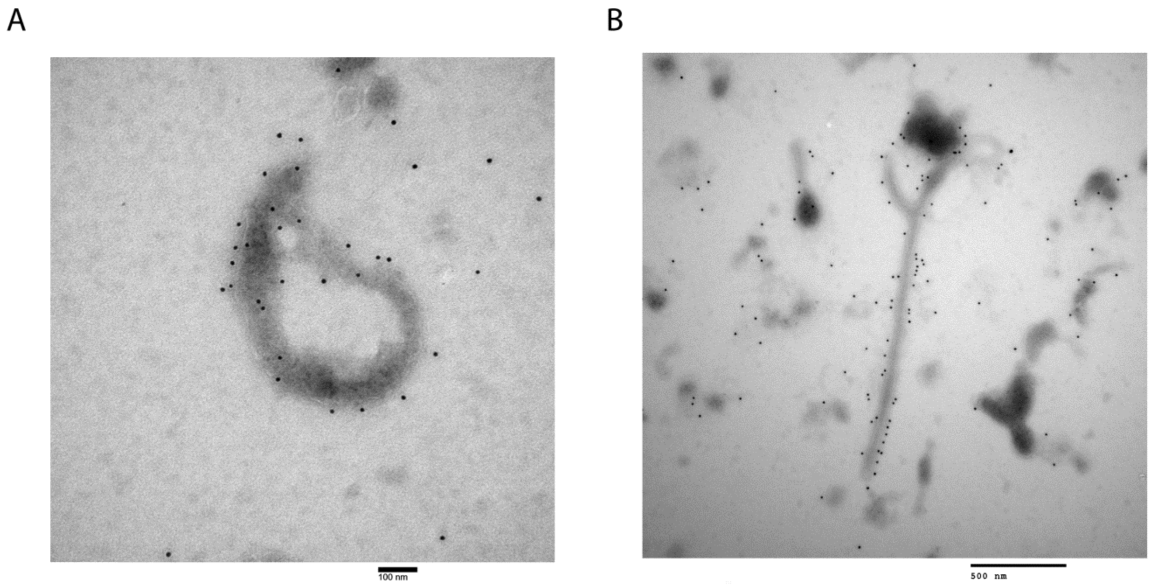

Figure S4. Electron micrographs of Nano-VLP with immunogold staining, performed using antibody 6D8.

Table S1. Survival of vaccinated mice after ma-Ebola challenge[1]

Experiment	Dose	Vaccination Materials	Survival	*p*-values[2]
**1**	10 µg	Control VLP	20 / 20	
		Heated, 37°C, 96 h	8 / 10	0.1518
		Heated 75°C, 15 min	1 / 10	**<.0001**
		Sonicated	16 / 20	0.3288
	2.5 µg	Control VLP	6 / 10	
		Heated 37°C, 96 h	6 / 10	0.9986
		Heated 75°C, 15 min	2 / 10	0.1700
		Sonicated	3 / 10	0.4040
**2**	10 µg	Control VLP	20 / 20	
		Sonicated and filtered, 0.45 µm	16 / 19	0.3796
		Sonicated and filtered, 0.8/0.2 µm	5 / 10	**0.0021**
		Sonicated	16 / 20	0.2418
	20 µg	Control VLP	10 / 10	
		Sonicated and filtered, 0.45 µm	10 / 10	1.0000
		Sonicated and filtered, 0.8/0.2 µm	8 / 10	0.1050
		Sonicated	10 / 10	1.0000

[1] None of the animals vaccinated with saline survived challenge (n=20).

[2] *p*-values were calculated using Fisher's exact tests to compare survival with Control VLP to each treatment group.

Table S2. Survival of mice vaccinated with Nano-VLP without adjuvant[1]

Dose	Vaccination Materials	Survival	*p*-values[2]
20 μg	Nano-VLP	6 / 10	**0.0029**
	Lyophilized Nano-VLP	10 / 10	**<.0001**
	Lyophilized and heated Nano-VLP	9 / 10	**<.0001**
5 μg	Nano-VLP	5 / 10	**0.0145**
	Lyophilized Nano-VLP	3 / 10	0.1463
	Lyophilized and heated Nano-VLP	7 / 10	**0.0004**

[1] None of the animals vaccinated with saline survived challenge (n=10).

[2] *p*-values compare survival of each treatment group vs. saline.
